# How social and geographical backgrounds affect hospital admission with a serious condition: a comparison of 11 immigrant groups with native-born Norwegians

**DOI:** 10.1186/s12913-018-3670-0

**Published:** 2018-11-08

**Authors:** Jon Erik Finnvold

**Affiliations:** Norwegian Social Research (NOVA), Oslo Metropolitan University, Postboks 4. St. Olavs plass, 0130 Oslo, Norway

**Keywords:** Immigration, Somatic hospital admissions, Socioeconomic position, Register study

## Abstract

**Background:**

The foreign-born population in Norway displays considerable diversity in terms of source country, socioeconomic status and settlement experience. This study assessed the consequences of this diversity for the risk of being admitted to hospital with a serious condition. To what extent could variations between immigrant and native-born hospitalisation patterns be accounted for by variations in income, education and residential area characteristics?

**Methods:**

The study linked information on socioeconomic and geographical level-of-living factors involving 2,820,283 individuals between 20 and 69 years old to hospital admissions recorded in Norway’s National Patient Registry. Immigrants from 11 of the most frequently represented countries were included. The outcome variable consisted of a selection of relatively serious diagnoses (neoplasms and endocrine, circulatory and respiratory diseases), totalling 548,140 admissions from 2008 to 2011. Age- and gender-adjusted admission rates were analysed using a Poisson regression.

**Results:**

The adjustments for income and education reduced the hospitalisation rates of almost all immigrant groups. The groups whose previous rates were above native-born rates moved towards the Norwegian reference, whereas groups that initially had lower age- and gender-adjusted rates compared with the Norwegian-born population increased the distance to the Norwegian reference. The risk of hospitalisation among most immigrant groups decreased compared with the Norwegian-born population when their income and educational levels were accounted for. Particularly, immigrants with lower levels of income or education tended to have relatively low hospitalisation rates, indicating the possibility of a healthy immigrant effect. While many immigrant groups used less somatic healthcare than the native-born population did, higher educational or income levels did not prevent hospitalisation to the same extent as they did for the native-born population.

**Conclusions:**

Although adjustments for socioeconomic factors tended towards lower hospitalisation rates for most immigrant groups, the adjustments did not reduce the considerable variations among individual countries.

## Background

Immigration to Norway has expanded rapidly; the population of immigrants (defined as foreign-born with foreign-born parents) increased from 2% of the total population in 1990 to 12% in 2014. This study assess immigrants’ risk of being hospitalised with a serious condition compared with the native-born population. Research in several European countries claims that immigrants encounter a double jeopardy at both individual and contextual levels [1–7]. The assumption seems to be that a substantial part of the poor health of immigrants relates to socioeconomic factors and unfavourable geographical environments. The immigration status might also add to the risk factors, such as low educational and income levels, consequently placing immigrants in a position of ‘cumulative disadvantage’ [8]. Additionally, immigrants often have jobs below their educational levels, causing social degradation and implying their lower likelihood of benefiting from a higher education compared with the native-born population.

On the other hand, it is possible to question the assumptions that immigrants in general have higher rates of healthcare utilisation and that their income, educational and residential patterns contribute to their vulnerability. In fact, several studies document the presence of a ‘healthy immigrant effect’, stating that foreign-born groups tend to have lower morbidity and mortality rates than those of native-born populations [9–12]. This effect can be accounted for partly by a selection of individuals who are healthy enough to migrate and partly by lower rates of risky behaviours among certain immigrant groups. A recent Norwegian study on morbidity finds higher mortality rates of 25–79-year-old native Norwegians than those of most immigrant groups, but immigrant women from Sub-Saharan Africa tend to have high mortality rates [13]. Specific studies have disputed the idea that social background is a significant factor contributing to poor health among immigrants [14]. The more educated immigrants are more likely to share cultural similarities with the host population and perhaps be in a better position to take advantage of what the healthcare system has to offer.

Furthermore, the association between socioeconomically deprived areas and negative health outcomes for immigrants can also be questioned, as some studies have identified possible beneficial effects of segregation, the so-called ‘ethnic density effect’ [15, 16]. It follows that even if immigrants settle in geographical areas with a high prevalence of health-related risk factors, it does not necessarily result in an increased risk of hospitalisation.

In a 2009 review of migrants’ utilisation of somatic healthcare services in Europe [17], the authors conclude that immigrants tend to have a lower level of use of preventive services, more contact with primary care and the same or higher level of use of specialist healthcare compared with the native-born population. A larger registry-based Norwegian study on emergency room contacts concludes that immigrants tend to have more often emergency visits, particularly those who arrived more recently [18]. Several studies on the use of general practice services have been conducted in Norway, with inconclusive results about whether immigrants more often consult general practitioners (GPs) [19, 20].

A few international studies have identified immigrants from specific countries and have applied measures of socioeconomic position, explicitly in relation to hospital utilisation [2, 21–23]. The last three cited studies [22–24] conducted surveys, whereas the first [2] based their analyses on registry data from Scotland, using a methodology similar to the present study. The Scottish study concludes that adjustments for socioeconomic variables have little effect on hospitalisation for cardiovascular diseases.

As few previous studies have addressed the effect of socioeconomic and spatial risk factors, it is difficult to formulate a specific expectation regarding the impact on immigrants’ hospitalisation rates in Norway. Given the diversity among different immigrant groups in Norway, a range of outcomes might also be expected with respect to hospitalization rates.

Health services in Norway are predominantly public, and utilisation of hospital services is free of charge. However, individuals cannot access specialist care without a referral from a GP, who acts as an access-regulating gatekeeper. General medical practice in Norway is organised as a patient-list system, implying that almost all inhabitants are registered with regular GPs. Immigration is marked by four main groups or causes; refugees, labour migrants, family reunion and educational purposes. It is mandatory for all immigrant groups to apply to The Norwegian Directorate of Immigration if they want to visit or live in Norway. There are no waiting or qualification periods to qualify for healthcare for immigrants, as they have the same right to healthcare as the rest of the population, even during the application period. The application criteria differ among the different causes for migration, although health condition and disability are not included in these criteria. A major present concern of the Norwegian immigration ministry relate to asylum seekers and documentation of the actual dangers they face in their country of birth.

This present study’s analyses are based on a dataset that merges data from Norway’s National Patient Registry (NPR) with data from sociodemographic registries for the first time in Norway. The comprehensive possibilities represented by Norwegian register data are used to raise two specific research questions:

- To what extent do demographic, socioeconomic and spatial risk factors increase or reduce differences in hospitalisation rates between immigrants and native-born Norwegians?
- Furthermore, do higher income or educational levels have a protective effect on the risk of being hospitalised for immigrants as well as for native-born Norwegians?

## Methods

### Design and setting

Immigrants, in this study, are defined as foreign-born people with foreign-born parents. The immigrant population is extremely diverse in terms of source country, socioeconomic status and settlement experience [24]. As specific immigrant groups vary in their social composition and geographical settlement patterns, it is important to identify each group on its own, instead of classifying the groups under heterogeneous categories. This study identified 11 of the most frequently represented immigrant countries in the analyses. Considerable variations also exist in terms of the period of arrival and the reasons for migrating. A couple of studies address an acculturation process, in which immigrants’ healthcare utilisation approaches the levels in their host country as they assimilate into the mainstream culture [25, 26]. For reasons such as education, work, family reunion or exile, migration might have different implications for healthcare utilisation patterns. Hence, these factors are controlled for in the analyses.

### Data

The outcome measure included all hospital admissions from 2008 to 2011 for the following diagnostic groups: neoplasms and endocrine, circulatory and respiratory diseases (International Classification of Diseases v.10 codes C00-D48, E00-E90, 100–199 and J00-J99, respectively). Of the four groups, circulatory diseases (42%) and neoplasms (34%) were the most frequent. The diagnostic groups were chosen because they represent relatively serious cases involving potentially life-threatening conditions, such as cardiovascular diseases and various types of cancer. The four diagnostic groups accounted for 72% of all deaths in the population under investigation over the four-year period, with the NPR providing the data. Owing to ethical considerations, only the main groups of diagnoses were permitted to be identified at the individual level.

The population under investigation included all Norwegian-born natives and immigrants from 11 countries, comprising Sweden, Poland, Vietnam, Russia, Somalia, Sri Lanka, Turkey, Bosnia, Iran, Iraq and Pakistan. As immigrants in general are younger than their native-born counterparts, the study population was limited to individuals from 20 to 69 years old, totalling 2,820,283 individuals, of whom the 11 immigrant groups comprised 4.1%.

Data on the country of birth, family income and education were added to the dataset by Statistics Norway and linked to the population through the use of personal identification numbers. Due to ethical considerations, the age information was only available for the researchers at 10-year intervals. The availability of identification numbers for the resident municipality and city districts (Oslo) made it possible to add pre-coded variables describing the local level of living conditions in the resident municipality/city region (percentage of the population with a university education, percentage of social assistance recipients and percentage of disability benefit recipients).

The information about education was missing in many cases for several immigrant groups, often due to their late arrival and the lack of registry updates [24]. The registry data did not include the reasons for the migration (education, work, family reunion or exile) of individuals who arrived before 1990 [27].

The outcome variable involved the count data – the number of admissions during a defined time period suitable for Poisson regression. Incidence-rate ratios were calculated by using multivariate analyses. Ethical approval was granted by the Norwegian Data Inspectorate, as well as by other relevant institutions and owners of specific parts of the data.

## Results

### Sociodemographics, settlement patterns and hospital admissions: Descriptive statistics

Table 1 presents the descriptive statistics on the individual countries included in the analyses. The age ranges significantly varied, thereby mirroring the immigration history of each group. The relatively young age range that characterised immigrants from countries such as Iran and Somalia reflected a relatively recent influx of mainly refugees, as well as job-seeking migrants from Poland. Other immigrant groups, such as Pakistanis, had an age range similar to that of native Norwegians. However, the oldest age group (60–69) was relatively predominant for Norwegians compared with all immigrant groups. Males were often over-represented among immigrants. Notable exceptions existed, such as the low percentage of male immigrants from Russia (27%) compared with that of Poland (70%), illustrating the difficulty in generalising among immigrant groups, even from the same region. Because the age and the gender demographics are important for healthcare utilisation, it is necessary to account for these variations in the analyses.

**Table 1:** Descriptive statistics for adult population 20–69 years, 01.01.2008 across 11 immigrant groups and native Norwegians. Gender, age, arrival in Norway, motive for migration, income and education Percent

	Country of birth											
	Norway	Sweden	Poland	Bosnia	Russia	Somalia	Turkey	Sri Lanka	Iraq	Iran	Pakistan	Vietnam
Gender												
Percent male	51	50	70	50	27	55	57	52	59	56	52	48
Age												
20–29	19	23	27	25	30	38	27	18	30	25	23	22
30–39	22	28	35	22	31	36	34	32	38	24	28	32
40–49	22	21	23	25	24	19	23	36	22	34	23	27
50–59	21	16	12	20	11	5	11	11	8	13	17	14
60–69	17	12	2	9	3	2	4	4	2	4	9	6
Total	100	100	100	100	100	100	100	100	100	100	100	100
Arrival in Norway												
Percentage that arrived in 2000 or later		36	81	9	66	50	23	15	40	24	17	12
Motive for migrating												
Labour	0	0	71	1	8	0	2	1	0	1	1	0
Family reunion	0	0	15	8	52	25	49	36	32	17	34	25
Refugee	0	0	0	86	25	66	2	17	61	39	2	17
Education	0	0	2	0	12	0	1	1	0	1	1	1
Other/not immigrant/missing	100	100	12	5	3	10	46	45	6	43	62	57
Total	100	100	100	100	100	100	100	100	100	100	100	100
Education												
Missing	1	24	41	5	16	22	15	13	18	8	17	10
Primary school	22	11	7	21	24	53	53	37	41	29	47	43
Secondary school	46	29	32	44	19	18	22	31	19	31	21	30
University level I	24	27	11	24	19	6	7	15	17	23	11	13
University level II	7	10	9	7	21	1	2	3	5	9	3	5
Total	100	100	100	100	100	100	100	100	100	100	100	100
Family income												
Less than mean 4th income quartile	24	24	51	31	37	73	40	23	56	44	31	33

The assumption is that all immigrants who have low income and educational levels have to be treated with caution. Returning to the contrast between Polish and Russian immigrants, the latter group had the highest educational attainment of all the groups, whereas the Poles had among the lowest (Table 1). Among non-European groups, Iraqi and Iranian immigrants’ educational levels were almost comparable to that of native-born Norwegians. As for income, considerable variations existed among the groups. Several non-Western immigrant groups’ income levels were significantly below that of native Norwegians; however, the generally high income level of immigrants from Sri Lanka pointed out the difficulty in making sweeping generalisations. When comparing groups, it is important to note that descriptive statistics can only provide crude measures, without any age and gender standardisations.

Regarding residential patterns (see Table 9, Appendix), several immigrant groups lived in areas whose residents had a relatively high educational level, such as Swedes, Somalis and Iranians, areas also characterised by a low number of disability benefit recipients, thus reflecting an urban or centralised settlement pattern. In contrast, areas with higher numbers of social assistance recipients had in many cases a considerable degree of immigrant clustering.

In total, 548,140 admissions were registered in the four-year study period (2008–2011), of which native-born Norwegians accounted for almost 97% (Table 2). Immigrant groups from five countries had lower age-adjusted rates compared with native-born Norwegians, those from three countries occupied a level above, while those from the remaining three countries had a level close to that of native-born Norwegians.

**Table 2:** Somatic hospital admissions, selection of diagnostic groups, 2008–2011 for adult population 20–69 years. Age-adjusted (as per 1.jan 2008) rates pr. 1000 person years

	Total	Percent	Age-adjusted (as per 1.jan 2008) rates pr. 1000 person years
Norway	531,207	96.91	51.7
Sweden	2722	0.50	42.8
Poland	1543	0.28	37.1
Bosnia	1619	0.30	51.1
Russia	654	0.12	36.2
Somalia	1029	0.19	45.1
Turkey	1149	0.21	52.5
Sri Lanka	955	0.17	53.4
Irak	1434	0.26	63.6
Iran	1462	0.27	55.8
Pakistan	3311	0.60	78.7
Vietnam	1055	0.19	33.1

### Changes in incidence-rate ratios

Table 3 displays the crude incidence rate ratios for groups with a different country of birth, using individuals born in Norway as a reference group. With the exception of immigrants from Pakistan, all of the groups had incidence ratios significantly below the Norwegian reference.

**Table 3:** Hospital admissions 2008–2011. Incidence rate ratios following poisson regression analysis. (95% C.I)

Norway (ref)	1	–
Sweden	0.74***	(0.70–0.77)
Poland	0.42***	(0.44–0.45)
Bosnia	0.79***	(0.75–0.83)
Russia	0.44***	(0.41–0.48)
Somalia	0.43***	(0.41–0.47)
Turkey	0.67***	(0.63–0.72)
Sri Lanka	0.67***	(0.63–0.72)
Iraq	0.61***	(0.58–0.65)
Iran	0.73***	(0.69–0.77)
Pakistan	1.23***	(1.18–1.28)
Vietnam	0.49***	(0.50–0.52)
Log Likelihood	− 177 8050	

*p < 0.05, **p < 0.01, ***p < 0.001

^1^Individuals with missing education was excluded from the analysis

Table 4 displays age- and gender-adjusted incidence-rate ratios. Due to the younger age structure of all immigrant groups compared to the native population, the incidence ratios displayed in Table 2 are considerably closer to the national average. Of the 11 countries, five had incidence ratios below the Norwegian reference (Sweden, Poland, Russia, Somalia and Vietnam). No differences were found in two countries (Turkey and Sri Lanka), whereas Iraq and Iran had slightly higher utilisation rates, and Pakistan showed significantly higher rates. In some cases, the differences were substantial. An adjusted incidence-rate ratio of 0.69 (Poland and Vietnam) indicated a utilisation level that was 45% below the Norwegian reference, while a rate of 1.49 (Pakistanis) showed a utilisation level that was 49% above the Norwegian reference.

**Table 4:** Hospital admissions 2008–2011. Incidence rate ratios following multivariate poisson regression analysis. Adjusted for age & gender. (95% C.I)

Norway (ref)	1	–
Sweden	0.78***	(0.75–0.81)
Poland	0.65***	(0.61–0.68)
Bosnia	0.97	(0.93–1.07)
Russia	0.79***	(0.73–0.86)
Somalia	0.91*	(0.85–0.97)
Turkey	1.03	(0.97–1.10)
Sri Lanka	1.02	(0.95–1.08)
Iraq	1.07*	(1.01–1.14)
Iran	1.07*	(1.01–1.12)
Pakistan	1.49***	(1.43–1.54)
Vietnam	0.69***	(0.65–0.73)
Log Likelihood	− 160 5285	

*p < 0.05, **p < 0.01, ***p < 0.001

Definition of variables: Gender: male = 1, female =2. Age: 20–29 = reference, additional four variables for each age group 30–39, 40–49, 50–59, 60–69. ^1^Individuals with missing education was excluded from the analysis

How were the estimates of incidence ratios affected by the inclusion of education and income variables in the analyses (Table 5)? The estimates showed that only immigrants from Pakistan had a level of utilisation above the Norwegian reference. Particularly, the estimates for Somali, Turkish and Vietnamese immigrants were markedly lower after adjustments. For Vietnamese immigrants, estimates were 72% lower than the Norwegian reference; the corresponding estimates for Somali and Turkish immigrants were 45 and 18% lower, respectively. In the majority of the cases, adjustments for education and income lowered the incidence ratios compared with the Norwegian reference.

**Table 5:** Hospital admissions 2008–2011. Incidence rate ratios following multivariate poisson regression analysis. Adjusted for age, gender, family income & educational attainments (95% C.I)

Norway (ref)	1	–
Sweden	0.86***	(0.82–0.90)
Poland	0.66***	(0.62–0.70)
Bosnia	0.93**	(0.88–0.98)
Russia	0.84***	(0.77–0.92)
Somalia	0.67***	(0.62–0.73)
Turkey	0.85***	(0.79–0.91)
Sri Lanka	1.00	(0.94–1.08)
Iraq	0.99	(0.93–1.05)
Iran	1.01	(0.95–1.07)
Pakistan	1.41***	(1.36–1.47)
Vietnam	0.58***	(0.54–0.63)
Log Likelihood	− 132 9928	

*p < 0.05, **p < 0.01, ***p < 0.001

Definition of variables: Gender: male = 1, female =2. Age: 20–29 = reference, additional four variables for each age group 30–39, 40–49, 50–59, 60–69. Family income: lowest incom quintile = reference, additional four variables for each quintile

Education: Primary education (1-10th grade) = reference, additional three variables with the highest completed educational leve, secondary education (10th -14th grade), lower level university education (14th – 17th grade) and higher level university education (more than 18 years)

^1^Individuals with missing education was excluded from the analysis

The rate ratios of Table 6 included variables related to level-of-living conditions in resident municipality, the date of arrival and the reason for migration. No significant effect of date of arrival could be observed (results not showed in table). The effect of work-based migration was evident in the cases of Polish and Russian immigrants. Controlling for these factors, Polish immigrants’ utilisation rates appeared to be comparable to the Norwegian reference. Moreover, the low rates for Russian immigrants disappeared when adjusting for reasons for migrating. However, although many immigrants from non-Western countries were refugees, controlling for this factor did not seem to alter the estimates, nor did arrival before or after the year 2000 influence the hospitalisation rates.

**Table 6:** Hospital admissions 2008–2011. Incidence rate ratios following poisson regression analysis. Adjusted for age, gender, family income, educational attainments, geographical location, reason for migrating and length of stay in Norway (95% C.I)

Norway (ref)	1	
Sweden	0.87***	(0.84–0.92)
Poland	1.02	(0.94–1.10)
Bosnia	0.93	(0.86–1.01)
Russia	1.02	(0.92–1.13)
Somalia	0.71***	(0.65–0.78)
Turkey	0.93*	(0.86–0.99)
Sri Lanka	1.08*	(1.00–1.16)
Iraq	1.03	(0.95–1.12)
Iran	1.06	(0.99–1.13)
Pakistan	1.50***	(1.44–1.57)
Vietnam	0.60***	(0.56–0.65)
Log Likelihood	−1,329,806	

*p < 0.05, **p < 0.01, ***p < 0.001

Definition of variables: Gender: male = 1, female =2. Age: 20–29 = reference, additional four variables for each age group 30–39, 40–49, 50–59, 60–69. Family income: lowest incom quintile = reference, additional four variables for each quintile

Education: Primary education (1-10th grade) = reference, additional three variables with the highest completed educational leve, secondary education (10th -14th grade), lower level university education (14th – 17th grade) and higher level university education (more than 18 years). Motive for migration: four variables: Labor migant/other, family reunion/other, asylum-seeker/other, education/other. Length of stay: Arrived after 2000/other. Four variables describing level-of-living conditions in resident municipality/city district (Oslo): More than 42,9% of population 20–66 years with a university level education/other, more than 5,5% of population 20–66 years recipients of social assistance/other, more than 7,5% of population 16–67 years recipients of disability benefit/other, level of unemployment more than 3,8% of adult population 20–66 years/other

^1^Individuals with missing education was excluded from the analysis

Overall, two general patterns can be observed in Tables 3, 4, 5 and 6 regarding changes in incidence ratios adjustments in Tables 5 and 6, compared to the age- and gender-adjusted rates in Table 4. For one group of countries, adjustments make moderate or marginal changes in the differences in incidence ratios, compared with the age- and gender-adjusted incidence ratios. This group includes Sweden, Sri Lanka, Iran, Iraq, Pakistan and Vietnam. A second group of countries includes those where inclusion of the new variables in Tables 5 and 6 leads to more noticeable changes; these are Poland, Bosnia, Russia, Somalia and Turkey. For the second group, model adjustments tended to lead towards rate ratios that approach the Norwegian reference category, or, in cases of Somalia and Turkey, where adjustments in Table 5 increase the discrepancy.

### Stratified analyses

Table 7 presents the results of the stratified analyses, in which the sample is divided into three sub-samples according to the educational level. To some extent, the findings revealed an overall tendency; specifically, the incidence ratios in the sample with primary education were significantly below the Norwegian reference in almost all cases. This pattern changed in the second and the third analyses of individuals with a secondary or tertiary education; the incidence ratios were either significantly higher than the Norwegian reference (Bosnia, Sri Lanka, Iran and Iraq), or no significant difference could be found (Poland, Russia, Somalia, Turkey and Vietnam). Two countries showed exceptions to this pattern; for Swedes, the rates were low in all of the analyses, and for Pakistanis, the rates were consistently above the Norwegian reference. Notably, however, in the case of Pakistani immigrants with a primary education, the incidence ratios were relatively close to the Norwegian reference.

**Table 7:** Hospital admissions 2008–2011. Age- and gender adjusted incidence rate ratios following multivariate poisson regression analysis. (95% C.I)

	Model 1 Primary education as highest completed education		Model 2 Secondary education as highest completed education (13–14 years)		Model 3 Tertiary/university- education (more than17 years)	
Norway (ref)	1	–	1	–		
Sweden	0,85***	(0,79 – 0,92)	0,84***	(0,78 – 0,90)	0,81***	(0,76 - 0,87)
Poland	0,60***	(0,52 - 0,69)	0,58***	(0,53 – 0,63)	0,93	(0,85 – 1,03)
Bosnia	0,85**	(0,77 – 0,93)	0,97	(0,90 – 1,05)	1,20***	(1,10 – 1,32)
Russia	0,58***	(0,50 - 0,67)	0,85	(0,73 – 1,00)	1,00	(0,89 – 1,13)
Somalia	0,62***	(0,57 – 0,68)	1,07	(0,93 – 1,23)	0,86	(0,68 – 1,09)
Turkey	0,81***	(0,75 – 0,87)	0,97	(0,84 – 1,13)	0,77	(0,60 – 1,00)
Sri Lanka	0,73***	(0,66 – 0,81)	1,10	(0,99 – 1,23)	1,19*	(1,02 – 1,40)
Iraq	0,84***	(0,78 – 0,91)	1,15*	(1,02 – 1,30)	1,14*	(1,02 – 1,27)
Iran	0,86**	(0,78 – 0,94)	1,26***	(1,15 – 1,37)	1,11*	(1,01 – 1,23)
Pakistan	1,17***	(1,12 – 1,23)	1,38***	(1,27 – 1,49)	1,67***	(1,52 – 1,82)
Vietnam	0,50***	(0,46 – 0,54)	0,67***	(0,59 – 0,75)	1,00	(0,86 – 1,15)

*p < 0.05, **p < 0.01, ***p < 0.001

Definition of variables: Gender: male = 1, female =2. Age: 20–29 = reference, additional four variables for each age group 30–39, 40–49, 50–59, 60–69

^1^Individuals with missing education was excluded from the analysis

The patterns described in Table 7 are mostly present in the analyses of income groups, as displayed in Table 8. Overall, immigrants in the low-income groups were significantly less likely to be hospitalised compared with their Norwegian-born counterparts. For the higher income groups, immigrants tended to be more often hospitalised or had hospitalisation rates that were not significantly different from the Norwegian reference. Exceptions emerged again (as above) for Swedes and Pakistanis. Another exception was found among Sri Lankans, for whom no differences in the hospitalisation rates of any of the income groups could be reported. It must also be noted that for Somali immigrants with higher income or educational levels, incidence ratios (not significant) were below the Norwegian reference.

**Table 8:** Hospital admissions 2008–2011. Age- and gender adjusted incidence rate ratios following multivariate poisson regression analysis. (95% C.I)

Income quintiles						
	Model 1 1 (lowest income)		Model 1 2–4 (intermediate income)		Model 1 5 (highest income)	
Norway (ref)	1	–	1	–	1	–
Sweden	0.76***	(0.68–0.84)	0.84***	(0.80–0.89)	0.76***	(0.66–0.88)
Poland	0.38***	(0.34–0.42)	0.67***	(0.63–0.71)	1.44**	(1.14–1.82)
Bosnia	0.74**	(0.67–0.83)	0.99	(0.92–1.05)	1.38*	(1.00–1.91)
Russia	0.53***	(0.44–0.64)	0.90*	(0.82–0.98)	1.17	(0.78–1.76)
Somalia	0.70***	(0.63–0.77)	0.97	(0.88–1.06)	0.37	(0.05–2.60)
Turkey	0.74***	(0.64–0.84)	1.06	(0.99–1.15)	1.49*	(1.01–2.20)
Sri Lanka	1.05	(0.91–1.22)	1.05	(0.97–1.13)	1.11	(0.73–1.69)
Iraq	0.78***	(0.71–0.87)	1.23***	(1.15–1.31)	1.28	(0.79–2.06)
Iran	0.84**	(0.76–0.93)	1.07*	(1.00–1.15)	0.96	(0.69–2.34)
Pakistan	1.21***	(1.11–1.33)	1.61***	(1.54–1.68)	2.10***	(1.82–2.44)
Vietnam	0.47***	(0.40–0.54)	0.68***	(0.63–0.74)	0.88	(0.62–1.25)

*p < 0.05, **p < 0.01, ***p < 0.001

Definition of variables: Gender: male = 1, female =2. Age: 20–29 = reference, additional four variables for each age group 30–39, 40–49, 50–59, 60–69

## Discussion

Due to their younger age structure, immigrants are, in general, less likely to be admitted to hospital for a serious health condition compared to the native population. Although several of the immigrant groups had lower age- and gender-adjusted hospitalisation rates compared with native Norwegians, the results displayed a great deal of variations. Pakistani immigrants experienced hospital admission levels that were significantly above those of the rest of the groups, including the Norwegian-born cohort. Iranian and Iraqi immigrants’ rates were slightly above the Norwegian reference, whereas immigrants from Vietnam and Poland in particular had hospitalisation rates that were considerably below that of native Norwegians.

When adjusting for income and education, some countries whose previous rates were above that of the native-born population moved towards the Norwegian reference; only Pakistani immigrants retained rates that were statistically significant above the reference. However, a significant group of countries did not experience marked changes in incidence ratios when adjusting for income and education, nor could the addition of further sociodemographic variables account for differences in incidence ratios among countries. For this group of countries, other factors not accounted for in the analyses override the impact of the sociodemographic variables included in the analyses.

The stratified analyses clearly indicate that compared with native Norwegians, immigrants with lower levels of education or income in most cases have a significantly lower probability of hospitalisation with a serious condition. The health risk associated with a lower income or educational attainment of the native-born cohort does not appear to be present at the same level for most immigrant groups, with the only exception being Pakistani immigrants. However, Pakistanis with low income and educational levels have an incidence-rate ratio that is considerably closer to the Norwegian-born reference (1.17 and 1.21, respectively) compared with Pakistanis with high educational or income levels (1.67 and 2.10, respectively).

Considering the possible health benefits of having an income or education in the upper levels, the relationship between immigrants and their native-born counterparts seems to be the opposite. Immigrants with higher levels of education or income are in many cases more often admitted to hospital. These findings might imply that immigrants with higher incomes or educational attainment represent a health policy concern. Nonetheless, it is not necessarily the case that second-generation immigrants, who mostly tend to follow the general educational patterns [24], will display the same hospitalisation patterns as those of their first-generation parents.

The fact that immigrants more often (but not always) only completed primary education or earn lower incomes and that most immigrant groups with such characteristics are less likely to be admitted to hospital implies that adjusting for income and education lowers the hospitalisation rates compared with the native-born population. This result is in line with the previously mentioned studies’ claim that immigrants’ poor health is partly related to their socioeconomic position [2, 3]. An important contributing factor to this finding is the significantly lower rate of hospitalisation among almost all immigrant groups. In their 1967 classic study, ‘The American Occupational Structure’, Blau and Duncan observe that migration ‘has in recent decades become increasingly effective as a selective mechanism by which the more able are channelled to places where their potential can be realized’ [8], p. 274]. The authors do not explicitly refer to intranational migration. Several studies conducted in different countries have documented that a possible selective mechanism might also apply to transnational migration [7–10], so it follows that one possible explanation of the results can be related to the existence of a healthy immigrant effect.

More importantly perhaps, it could be argued that immigrants with lower levels of income or education differ from native Norwegians in ways that could help explain the lower incidence ratios. For native-born Norwegians, the lack of success in the education and labour market might involve a chronic disease or disability acquired from birth or early childhood. Similar life courses are less likely for immigrants with low levels of income or education, as a migrant without a secondary education could be thought of as a special case of ‘the healthy immigrant effect’. Nonetheless, it must again be emphasised that the healthy immigrant effect does not seem to apply to immigrants with higher levels of income or education.

In light of the findings in the epidemiological literature, the documented utilisation levels are in some cases expected (Pakistani immigrants) but unexpected in other cases. A significantly high risk of diabetes is found for non-European immigrants in both the United Kingdom and the Netherlands, while a literature review of the prevalence of diabetes in the Nordic countries suggests that diabetes is more frequent among non-European immigrant groups [5]. A Norwegian study on the prevalence of cardiovascular disease reports significantly higher rates among immigrants from Sri Lanka, Pakistan, Iran, Vietnam and Turkey [28]. Based on tests of blood pressure and cholesterol values, another study records a higher cardiovascular risk among immigrants from the same countries, with the exception of Vietnamese immigrants [29]. High rates of obesity are also found among immigrants from non-Western countries, again with the exception of Vietnam [30]. A 2010 research review about the public health challenge of immigrants in Norway concludes that they generally present with poor health conditions and are disproportionately exposed to a number of lifestyle- and diet-related illnesses and infectious diseases [31].

The inclusion of variables that involve measures of the level of living conditions in residential areas has in most cases no impact on the hospital admission rates. As observed in a couple of studies, immigrant-segregated settlement patterns could have positive health-related influences that might counter the possible negative effects of poor living conditions [13, 14]. Furthermore, the findings presented in this study only partially support the belief that immigrants generally live in socioeconomically deprived areas. Several immigrant groups also have a relatively geographically dispersed settlement pattern.

### Strengths and limitations

This study presents a major national investigation into variations in hospitalisation affecting a number of specific immigrant groups, including a substantial number of available hospital admissions. The Norwegian-born population is used as a reference category. This does not mean that this cohort’s level of utilisation represents a norm or in any way a measure of how often a group of individuals should be admitted to hospital. Although plausible explanations for the relatively low admission rates among several immigrant groups are suggested, underutilisation cannot be ruled out as a possibility. The patterns observed in the analyses do not necessarily reflect the real incidence of diseases with the selected diagnostic criteria. Without relevant measures of morbidity for the specific diseases in question, it is not easy to arrive at firm conclusions on whether the level of hospital utilisation of each immigrant group actually reflects the need for healthcare services. As observed by studies on medical sociology in general [32], as well as specific studies on immigrant utilisation of healthcare [33], decisions concerning illnesses may be informed by lay consultants, and people’s differences in their knowledge about diseases and sources of treatment might have a bearing on the patterns of utilisation documented in the present study. However, the adopted strategy to select diagnoses that all represent serious conditions to some extent counters the lack of information about morbidity, health profiles and other determinants of utilisation that are not directly related to health. Because the outcome only includes serious conditions, it could be expected that admission rates would reflect variations in morbidity, perhaps more so compared with the use of other sources of healthcare, such as outpatient services, emergency room utilisation, primary physicians or preventive health services.

The study is not necessarily applicable to other European countries. Educational attainment and income are key components in the analyses, and these are variables that may have a different impact in countries with a welfare state based on liberal or corporatist-statist models. The composition of immigrant groups may also differ from country to country. In addition, countries’ different policies regarding immigration may introduce health-related biases.

Immigrants from the same country might differ in terms of ethnic and cultural background and language, all factors with possible implications for health condition and healthcare-seeking strategies. This study used country of birth to define immigrants, limiting the possibility of analysing within-country variations.

Missing values for education in particular could also introduce bias, since missing values are relatively frequent among more recent immigrants [24].

## Conclusion

The general assumption that immigrants impose a burden on somatic healthcare institutions is not supported by this study’s findings, as hospitalisation rates substantially vary among immigrants from different countries, as well as between immigrants and native-born Norwegians. The implication of these results is that in the context of Norway, more heterogeneous models for explaining the impact of immigrants on the healthcare system are required. It is not the case that adjustments for socioeconomic factors always equal levels of hospitalisation, hence making immigrants more similar to the native-born population. The risk of hospitalisation associated with the lower levels of income or education observed for the native-born cohort does not seem to be present at the same level among most immigrant groups, with the exception of Pakistani immigrants. However, a higher level of education or income is less likely to protect immigrants from hospitalisation compared with their native-born counterparts.

Although in many cases, adjustments for socioeconomic factors lower hospitalisation rates, such adjustments offer little explanation for these differences. Further research to unveil the mechanisms behind the major differences among specific immigrant groups presents itself as an interesting agenda, especially in contrast to two of the most frequently represented immigrant groups with relatively long histories of immigration in Norway – the Vietnamese, with consistently low hospitalisation rates, and the Pakistanis, with consistently high hospitalisation rates. Research that addresses the experiences of specific immigrant groups has the potential to acquire new knowledge about the mechanisms that either prevent or cause costly hospital admissions, with serious implications for the well-being of individuals at risk.

## Appendix

**Table 9:** Descriptive statistics for adult population 20–69 years, 01.01.2008 across 11 immigrant groups and native Norwegians. Indicators of level-of-living conditions in resident municipality/city district (Oslo). Per cent

	Country of birth											
	Norway	Sweden	Poland	Bosnia	Russia	Somalia	Turkey	Sri Lanka	Iraq	Iran	Pakistan	Vietnam
Indicators of level-of-living conditions in resident municipality/city district:												
More than 42,9% of adult population 20–66 years with a university level education	7	22	12	7	11	13	6	5	7	15	7	6
More than 5,5% of adult population 20 66 years recipients of social assistance	8	12	9	10	11	30	19	28	16	15	33	16
More than 7,5% of adult population 16–67 years recipients of disability benefit	35	25	23	33	32	23	16	16	29	20	16	23
Level of unemployment more than 3,8% of adult population 20–66 years	2	5	6	5	5	20	16	29	11	11	37	13
(N)	2,681,182	21,361	24,064	10,352	8070	10,972	8642	7437	12,245	10,569	14,060	11,329

## Abbreviations

GP: General practitioner
NPR: Norwegian Patient Registry
US: United States
